# Alterations in Vitamin D signalling and metabolic pathways in breast cancer progression: a study of VDR, CYP27B1 and CYP24A1 expression in benign and malignant breast lesions Vitamin D pathways unbalanced in breast lesions

**DOI:** 10.1186/1471-2407-10-483

**Published:** 2010-09-11

**Authors:** Nair Lopes, Bárbara Sousa, Diana Martins, Madalena Gomes, Daniella Vieira, Luiz A Veronese, Fernanda Milanezi, Joana Paredes, José L Costa, Fernando Schmitt

**Affiliations:** 1 IPATIMUP - Institute of Molecular Pathology and Immunology of the University of Porto (Rua Dr Roberto Frias, s/n), Porto (4200-465), Portugal; 2 Department of Pathology, Federal University of Santa Catarina (Campus Reitor João David Ferreira Lima), Florianópolis (88040-970), Brazil; 3 Department of Pathology, General Hospital of UNIMED (Rua Gaspar de Lemos, 2), Araçatuba (16013-800), Brazil; 4 Medical Faculty of the University of Porto (Alameda Prof. Hernâni Monteiro), Porto (4200-319), Portugal

## Abstract

**Background:**

Breast cancer is a heterogeneous disease associated with different patient prognosis and responses to therapy. Vitamin D has been emerging as a potential treatment for cancer, as it has been demonstrated that it modulates proliferation, apoptosis, invasion and metastasis, among others. It acts mostly through the Vitamin D receptor (VDR) and the synthesis and degradation of this hormone are regulated by the enzymes CYP27B1 and CYP24A1, respectively. We aimed to study the expression of these three proteins by immunohistochemistry in a series of breast lesions.

**Methods:**

We have used a cohort comprising normal breast, benign mammary lesions, carcinomas *in situ *and invasive carcinomas and assessed the expression of the VDR, CYP27B1 and CYP24A1 by immunohistochemistry.

**Results:**

The results that we have obtained show that all proteins are expressed in the various breast tissues, although at different amounts. The VDR was frequently expressed in benign lesions (93.5%) and its levels of expression were diminished in invasive tumours (56.2%). Additionally, the VDR was strongly associated with the oestrogen receptor positivity in breast carcinomas. CYP27B1 expression is slightly lower in invasive carcinomas (44.6%) than in benign lesions (55.8%). In contrast, CYP24A1 expression was augmented in carcinomas (56.0% in *in situ *and 53.7% in invasive carcinomas) when compared with that in benign lesions (19.0%).

**Conclusions:**

From this study, we conclude that there is a deregulation of the Vitamin D signalling and metabolic pathways in breast cancer, favouring tumour progression. Thus, during mammary malignant transformation, tumour cells lose their ability to synthesize the active form of Vitamin D and respond to VDR-mediated Vitamin D effects, while increasing their ability to degrade this hormone.

## Background

Breast cancer is one of the major causes of death by cancer in women worldwide [1]. Nowadays, breast cancer is no longer considered to be a single disease, but is rather comprised of distinct tumour subtypes displaying different clinical outcomes [2]. Over the lifetime of the individual, in order to a tumour to develop it needs a combination of low-penetrance genetic factors and environmental aspects. Ultimately, cancer results from alterations in the control of the complex balance of proliferation, differentiation and programmed cell death [3] and these processes appear to be regulated by intrinsic and extrinsic factors, like niche signals, hormonal and dietary aspects, among others [4], [5].

Vitamin D is a lipid soluble substance that belongs to the family of secosteroid hormones. Its physiological role has been classically associated with calcium regulation and phosphate transport in bone metabolism. Apart from this endocrine role, subsequent studies have widened the range of functions for Vitamin D and this has been particularly important in the field of cancer research. Several authors have demonstrated, in various models of cancer (including the breast), the ability of Vitamin D to perform autocrine and paracrine functions. Specifically, it has been demonstrated the capacity to modulate cancer features, namely proliferation and differentiation [6], apoptosis [7], angiogenesis [8], invasion and metastasis [9].

Vitamin D exerts most of its biological activities by binding to a specific high-affinity receptor, the Vitamin D Receptor (VDR), that was first identified in a breast cancer cell line in 1979 [10]. The VDR belongs to the superfamily of nuclear receptors for steroid hormones and regulates gene expression by acting as a ligand-activated transcription factor [11]. Several studies have demonstrated that the VDR knockout mice display a higher incidence rate of carcinogen-induced preneoplastic breast lesions when compared with their littermates [12], [13]. These reports highlight the importance of the VDR deficiency in sensitizing the mammary gland to transformation in response to a carcinogenic agent. Immunohistochemical studies have confirmed that the VDR is expressed in samples from normal breast tissues [14] and also in breast cancer biopsy specimens [15]. Because the VDR is expressed in the mammary gland and Vitamin D has been shown to display anticarcinogenic properties, this hormone has emerged as a promising targeted therapy. But in order to keep the homeostasis of the organism the amount of circulating Vitamin D has to be tightly regulated. This is a very complex process, in which the main components are the enzymes 1α-hydroxylase/CYP27B1 (encoded by the gene *CYP27B1*) and 24-hydroxylase/CYP24A1 (encoded by the gene *CYP24A1*). CYP27B1 is responsible for the synthesis of the biologically active form of Vitamin D (1,25-dihydroxyvitamin D), whereas CYP24A1 mediates the catabolism of Vitamin D [16]. Several studies have focused their attention in the comparison of the levels of these enzymes in normal and tumour tissue. It has been observed that both CYP27B1 and CYP24A1 are up-regulated in breast tumours when compared with normal tissue. However, deregulated expression of CYP24A1 seems to abrogate the effects of CYP27B1, resulting in the degradation of Vitamin D to less active metabolites [17]. In contrast, a recent paper has demonstrated that CYP27B1 mRNA in breast tumours is decreased in comparison with normal mammary tissue [18]. Despite these findings, no reports regarding the expression by immunohistochemistry of the VDR, CYP27B1 and CYP24A1 in the mammary gland have been described. The main purpose of this work was to perform an immunohistochemical study of the expression of the VDR, CYP27B1 and CYP24A1 in a comprehensive series of human breast tissues comprised of normal breast, benign mammary lesions, carcinomas *in situ *and invasive breast carcinomas.

## Methods

### Patient's selection and Tissue Microarray construction

We have studied a cohort of 379 benign lesion samples and 189 cases of carcinomas *in situ*, collected from the archives of the Pathology Department of General Hospital of UNIMED in Araçatuba, Brazil. Three hundred and fifty cases of invasive breast carcinomas were retrieved from the archives of the Pathology Department of the Federal University of Santa Catarina, Florianópolis, Brazil (161 cases) and from the Pathology Department of General Hospital of UNIMED in Araçatuba, Brazil (189 tumour samples). This last series of 189 invasive carcinomas contains, in the same block, the aforementioned carcinomas *in situ*. Additionally, 29 cases of normal breast tissue were included in the study. The normal breast tissue, carcinomas *in situ *and invasive tumour samples were collected between 1994 and 2004. The series of benign lesions was collected between 2002 and 2006.

Representative areas of the different lesions were carefully selected on the H&E-stained sections, by 2 pathologists (DV and LAV) and marked on individual paraffin blocks. Two tissue cores (2 mm in diameter) were obtained from each selected specimen and precisely deposited into a recipient paraffin block using a TMA (Tissue Microarray) workstation (TMA builder, LabVision Corporation, USA). Several TMA blocks were constructed (40 for the invasive breast carcinomas, 22 for the carcinomas *in situ *and 17 for the benign lesions), each containing 24 tissue cores, arranged in a 4×6 sector. In each TMA block, at least 3 nonneoplastic breast tissue cores were also included as controls and 1 core of a non-breast sample (we have used testicular and liver tissues). To homogenize the paraffin of the receptor block and the paraffin of the cores extracted from the donor blocks, the TMAs were kept at 37°C for 3 hours. After construction, 2-μm tissue sections were cut and adhered to Superfrost Plus glass slides. An H&E-stained section from each block was reviewed to confirm the presence of morphological representative areas of the original lesions.

The present study has been conducted under the national regulative law for the usage of biological specimens from tumour banks, where the samples are exclusively available for research purposes in the case of retrospective studies.

### Immunohistochemistry

Immunohistochemical staining for Oestrogen Receptor (ER), HER2 and CK5 (Cytokeratin 5) was performed using the streptavidin-biotin-peroxidase technique (LabVision Corporation) in each set of glass slides comprising the TMAs, whereas P-cadherin (P-cad), EGFR (Epidermal Growth Factor Receptor) and Progesterone Receptor (PgR) used the HRP labelled polymer (DakoCytomation, USA) as described elsewhere [19]. Antigen unmasking for VDR was performed using a solution of pepsin A (4 g/L; Sigma-Aldrich) for 30 minutes at 37°C. Epitope retrieval for CYP27B1 and CYP24A1 was performed using a dilution of 1:100 of citrate buffer, pH = 6.0 (Vector Laboratories, Burlingame, CA, USA) at 98°C for 30 minutes. The antigen retrieval times, antibodies, dilutions and suppliers are listed in Table 1. Primary antibody incubation was performed overnight at 4°C for VDR and CYP24A1 and for 1 h at room temperature for CYP27B1. After washes, the slides were incubated with secondary antibody associated with HRP labelled polymer (ImmunoLogic, The Netherlands) for VDR or incubated with biotinylated secondary antibody (Santa Cruz, USA) followed by streptavidin-conjugated peroxidase (Labvision) during 15 min for CYP24A1 and CYP27B1, and immediately revealed with DAB (DakoCytomation). Tissues were then counterstained with Mayer's haematoxylin, dehydrated and cover-slipped using a permanent mounting solution (Zymed, USA). Positive and negative controls were included in each run in order to guarantee the reliability of the assays. Paraffin sections of a basal cell carcinoma of the skin, normal colon and normal liver were used as positive controls for VDR, CYP27B1 and CYP24A1 expression, respectively.

**Table 1:** Sources and dilutions of primary antibodies related to the Vitamin D metabolism used in this study for immunohistochemistry

Antibody	Clone	Manufacturer	Time of incubation (min)	Dilution	Antigen retrieval (min)
VDR	9A7γE10.4	Calbiochem, Germany	overnight	1:50	30

CYP27B1	C12	Santa Cruz, USA	60	1:200	30

CYP24A1	C18	Santa Cruz, USA	overnight	1:75	30

### Scoring and statistical analysis

The evaluation of the immunohistochemical results was performed by three pathologists (FS, FM and LAV). VDR nuclear expression was evaluated using the H-score method: intensity ranked from 1 to 3 (1 - weak, 2 - moderate, 3 - strong), and extension ranked from 1 to 10 (1 - 0-10% cells, 2 - 11-20% cells and so on, until a maximum score of 10) [20]. The scores for intensity and extension were multiplied and the following criterion was applied: the cases were considered negative when ranging from 1 to 4; samples ranking from 5 to 30 were considered to be positive. Considering the lack of previous reports for the immunohistochemical evaluation of the CYP27B1 and CYP24A1, we considered the cases to be positive only when cytoplasmic staining was observed. The other markers were scored as described in previous studies from our group [19], [21].

The Statview 5.0 software package (SAS Institute, USA) was used for all statistical analysis. Correlations between discrete variables were performed using the chi-square test and analysis of variance was employed to search for associations between continuous and discrete variables. In all analyses, a p value < 0.05 was considered to be statistically significant.

### Cell culture and Western blotting

MDA-MB-231 breast cancer cells were grown in complete Dulbecco's Modified Eagle Medium (DMEM) in the presence of 10% foetal bovine serum (Invitrogen, USA). Treatments with Vitamin D 100 nM (Cayman Chemical, USA) and ethanol (vehicle) were performed for 72 h, while the treatment with PTH (Parathyroid Hormone) (Sigma-Aldrich, Germany) 100 nM and water (vehicle) were performed for 4 h. Total cell lysates were obtained and the samples were separated in an SDS-polyacrylamide gel. After blotting into a nitrocellulose membrane (GE Healthcare Life Sciences, UK), staining for CYP27B1 and CYP24A1 was performed using the antibodies (Santa Cruz, USA) presented on Table 1 overnight at a dilution of 1:200. After washes, the membranes were incubated with a mouse anti-goat HRP secondary antibody (Santa Cruz) and were revealed with ECL (GE Healthcare Life Sciences).

### RNA extraction and Real-time PCR

RNA was extracted from formalin-fixed paraffin-embedded breast lesions using the RecoverAll Total Nucleic Acid Isolation Kit (Ambion, USA), according to the manufacturer's protocol. After extraction, RNA was quantified using NanoDrop spectrophotometer (Thermo Scientific, USA). cDNA was synthesized using the Omniscript Reverse Transcription kit (Qiagen, Germany) following the manufacturer's instructions. Finally, real-time PCR was performed using TaqMan Gene Expression Assays (Applied Biosystems, USA), using 2 mL of cDNA and in accordance to the manufacturer's protocol. The TaqMan Gene Expression Assays used were Hs00172113_m1 (VDR), Hs00168017_m1 (CYP27B1) and Hs00167999_m1 (CYP24A1). Reactions were performed using standard cycle parameters on an ABI PRISM Sequence 7000 Detection System (Applied Biosystems). Relative transcript levels were determined using Human GAPDH Endogenous Control (Applied Biosystems) as an internal reference. Differences between the breast tissue samples were determined using comparative delta C_T _method [22]. All reactions were done in triplicate and expressed as mean of the values from three separate experiments.

## Results

### VDR, CYP27B1 and CYP24A1 immunohistochemical staining

**Figure 1:**
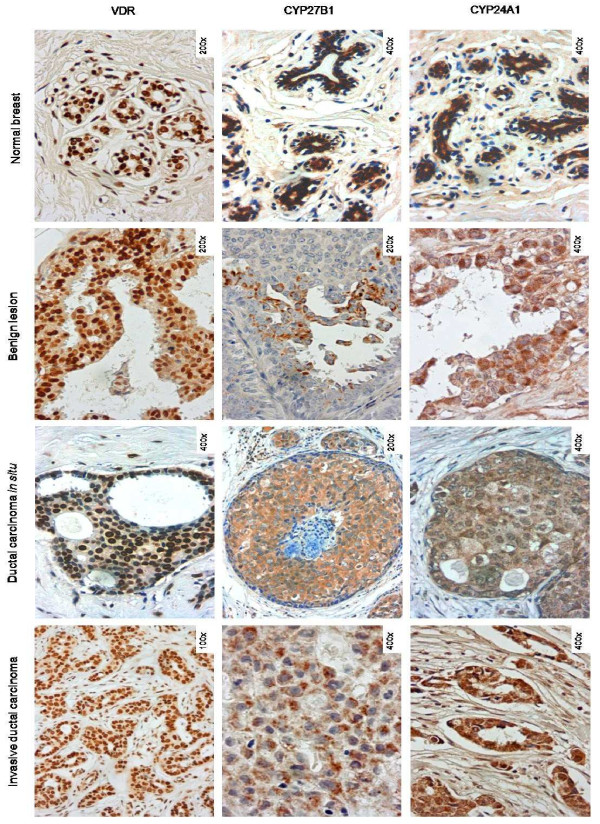
Immunohistochemical staining for the VDR, CYP27B1 and CYP24A1 in the different types of breast tissue

The expression patterns of the VDR, CYP27B1 and CYP24A1 have been evaluated by immunohistochemistry in 947 breast tissue samples arranged in 79 TMAs. From this set of cases, some samples could not be assessed due to the fact that either the core had fallen out or it did not have enough biological material to study. In all TMAs, positive and negative cases were obtained for each protein. The immunostainings for these markers had been previously validated in whole tissue sections with an overall agreement of 90%. A panel with representative immunostainings for each protein in different breast tissues is shown in Figure 1. We have observed that the VDR displays nuclear staining, as would be expected from a nuclear receptor which acts as a transcription factor. Considering CYP27B1 and CYP24A1 expression, nothing has ever been described on their expression status in the mammary gland, as far as we know. This is the first report showing the expression of these two enzymes in breast lesions. These proteins present cytoplasmic and granular staining, which could reflect their mitochondrial localisation. All proteins (VDR, CYP27B1 and CYP24A1) have been found to be expressed in all lesions studied and also in the normal breast tissue, although at different levels.

The differential expression of CYP27B1 and CYP24A1 was technically validated. MDA-MB-231 breast cancer cells have been treated with PTH 100 nM and Vitamin D 100 nM and total cell lysates have been extracted. Western blotting analysis has confirmed the expression of CYP27B1 and CYP24A1 upon treatment with the aforementioned hormones (Additional file 1: Figure S1). Additionally, using a group of randomly selected tissue samples, RNA was isolated and used in real-time PCR to confirm the immunohistochemical results (Additional file 2: Table S1). Our results have shown that positive cases in the TMAs displayed cDNA amplification in the real-time PCR and the opposite situation was observed for cases where no staining was present in the TMAs.

### Expression of the VDR, CYP27B1 and CYP24A1 in benign lesions of the mammary gland

In order to study the VDR, CYP27B1 and CYP24A1 expression in benign lesions of the mammary gland, we have evaluated 379 cases arranged in 17 TMAs. The series consisted of a variety of breast lesions, namely usual and atypical ductal hyperplasias (UDH represent 20.1%, corresponding to 76 samples; while ADH represent 5.4%, corresponding to 21 samples), columnar cell lesions (CCL - 25.6% of cases, corresponding to 97 samples), papillomatosis (16.9% of cases, corresponding to 64 samples) and adenosis (17.2% of cases, corresponding to 65 samples). The percentage of immunoreactive cases for the VDR was very high (93.5%, corresponding to 259 cases out of 277). Regarding the expression of CYP27B1, we have observed 55.8% of positive cases, corresponding to 173 lesions out of 310. Concerning CYP24A1 expression, we have detected 62 positive cases out of 327 samples (19.0%). Amongst all lesions, ADH cases were overall less immunoreactive to the three proteins.

We have correlated the histological classification of the benign lesions with the VDR, CYP27B1 and CYP24A1 expression, but no significant associations have been found (see Table 2 for further details).

**Table 2:** VDR, CYP27B1 and CYP24A1 expression in the various types of benign breast lesions

	VDR		CYP27B1		CYP24A1	
	+ (%)	- (%)	+ (%)	- (%)	+ (%)	- (%)

Usual ductal hyperplasia	84 (92.3)	7 (7.7)	57 (55.9)	45 (44.1)	23 (20.5)	89 (79.5)

Atypical ductal hyperplasia	9 (81.8)	2 (18.2)	4 (36.4)	7 (63.6)	1 (7.1)	13 (92.9)

Columnar cell lesions	63 (95.5)	3 (4.5)	43 (55.8)	34 (44.2)	13 (16.5)	66 (83.5)

Papillomatosis	45 (95.7)	2 (4.3)	30 (56.6)	23 (43.4)	9 (17.0)	44 (83.0)

Adenosis	49 (92.5)	4 (7.5)	32 (55.2)	26 (44.8)	13 (22.0)	46 (78)

**p value**	**0.4847**		**0.7994**		**0.6842**	

### Expression of the VDR, CYP27B1 and CYP24A1 in breast carcinomas *in situ*

A fully characterized series of 189 breast carcinomas *in situ *arranged in 22 TMAs was assessed for the expression patterns of VDR, CYP27B1 and CYP24A1. For the VDR, we have observed that 62 cases out of 131 cases (47.3%) displayed staining for this protein. Concerning CYP27B1 expression, we have encountered positive staining in 66.4% of the cases (91 out of 137 samples); whereas CYP24A1 expression was observed in 56.0% of the tumours (70 out of 125 cases).

We have also assessed the expression of other breast cancer biomarkers in our cohort (ER, HER2 and PgR and basal markers as defined by our group [19] and others [23]) and looked for the existence of correlations between the expression of the Vitamin D partners and these molecular markers (Table 3). ER expression has been observed in 117 cases (61.9%), HER2 protein was present in 37 cases (15.6%) and PgR expression was detected in 90 cases (47.6%). We have also tested our series for basal markers and have obtained the following results: EGFR expression is present in 10 cases (5.3%), CK5 is positive in 15 cases (7.9%) and P-cadherin was observed in 36 samples (19.0%). Expression of the VDR correlated positively with ER status (p = 0.0227), with a higher percentage of VDR-positive cases among the ER-positive tumours - 74.2% (46 out of 62 cases). Additionally, we have seen that there is an inverse correlation between the expression of the VDR and P-cadherin (p = 0.0078). CYP27B1 expression only presented an inverse correlation (p = 0.0295) with EGFR expression, but the number of cases positive for EGFR was very low. No statistically significant associations have been observed between CYP24A1 expression and the markers studied.

**Table 3:** VDR, CYP27B1 and CYP24A1 and other breast cancer biomarkers expression in carcinomas *in situ*

		VDR		CYP27B1		CYP24A1	
	
		+ (%)	- (%)	+ (%)	- (%)	+ (%)	- (%)
ER	+ (%)	46 (35.1)	38 (29.0)	58 (42.3)	29 (21.2)	41 (32.8)	36 (28.8)
	- (%)	16 (12.2)	31 (23.7)	33 (24.1)	17 (12.4)	29 (23.2)	19 (15.2)
	
	**p value**	**0.0227**		**ns**		**ns**	

HER2	+ (%)	9 (6.9)	14 (10.7)	18 (13.1)	7 (5.1)	9 (7.2)	12 (9.6)
	- (%)	53 (40.5)	55 (42.0)	73 (53.3)	39 (28.5)	61 (48.8)	43 (34.4)
	
	**p value**	**ns**		**ns**		**ns**	

PgR	+ (%)	35 (26.7)	30 (22.9)	49 (35.8)	18 (13.1)	38 (30.4)	22 (17.6)
	- (%)	27 (20.6)	39 (29.8)	42 (30.7)	28 (20.4)	32 (25.6)	33 (26.4)
	
	**p value**	**ns**		**ns**		**ns**	

CK5	+ (%)	3 (2.3)	8 (6.1)	7 (5.1)	4 (2.9)	8 (6.4)	4 (3.2)
	- (%)	59 (45.0)	61 (46.6)	84 (61.3)	42 (30.7)	62 (49.6)	51 (40.8)
	
	**p value**	**ns**		**ns**		**ns**	

EGFR	+ (%)	1 (0.8)	5 (3.8)	2 (1.5)	5 (3.7)	5 (4.0)	3 (2.4)
	- (%)	61 (46.6)	64 (48.9)	89 (65.0)	41 (29.9)	65 (52.0)	52 (41.6)
	
	**p value**	**ns**		**0.0295**		**ns**	

P-cad	+ (%)	4 (3.1)	16 (12.2)	14 (10.2)	12 (8.8)	16 (12.8)	7 (5.6)
	- (%)	58 (44.3)	53 (40.5)	77 (56.2)	34 (24.8)	54 (43.2)	48 (38.4)
	
	**p value**	**0.0078**		**ns**		**ns**	

ns: not significant.

### Expression of the VDR, CYP27B1 and CYP24A1 in invasive mammary carcinomas

We have evaluated 350 cases of invasive breast carcinomas arranged in 40 TMAs. The cohort corresponds to 189 cases of the series for which there was an *in situ *component in the adjacent area of the invasive tumour and an additional series of 161 cases of invasive breast carcinomas. Positive staining for the VDR has been observed in 56.2% of the cases (172 out of 306 cases). Regarding CYP27B1 expression, 44.6% of cases were positive (123 out of 276 samples), whereas 53.7% of cases (151 out of 281 tumours) presented positivity for CYP24A1.

Next, we searched for associations between the expression of Vitamin D partners and the expression of the molecular markers mentioned in the previous section (Table 4). We have obtained 197 cases (56.3%) positive for ER, 70 cases (20%) for HER2 and 143 cases (40.9%) for PgR. As for basal markers, we have observed that 13 cases (3.7%) were positive for EGFR expression, 48 cases (13.7%) presented positivity for CK5 and 93 cases (26.6%) stained for P-cadherin.

**Table 4:** VDR, CYP27B1 and CYP24A1 and other breast cancer biomarkers expression in invasive breast tumours

		VDR		CYP27B1		CYP24A1	
	
		+ (%)	- (%)	+ (%)	- (%)	+ (%)	- (%)
ER	+ (%)	114 (37.3)	60 (19.6)	70 (25.4)	86 (31.2)	93 (33.1)	66 (23.5)
	- (%)	58 (19.0)	74 (24.2)	53 (19.2)	67 (24.3)	58 (20.6)	64 (22.8)
	
	**p value**	**0.0002**		**ns**		**ns**	

HER2	+ (%)	26 (8.6)	34 (11.3)	31 (11.4)	25 (9.2)	29 (10.4)	30 (10.8)
	- (%)	144 (47.7)	98 (32.5)	90 (33.1)	126 (46.3)	121 (43.5)	98 (35.3)
	
	**p value**	**0.0238**		**ns**		**ns**	

PgR	+ (%)	71 (23.3)	59 (19.3)	52 (18.8)	64 (23.2)	71 (25.3)	46 (16.4)
	- (%)	100 (32.8)	75 (24.6)	71 (25.7)	89 (32.2)	80 (28.5)	84 (29.9)
	
	**p value**	**ns**		**ns**		**0.0485**	

CK5	+ (%)	27 (8.8)	19 (6.2)	15 (5.4)	24 (8.7)	27 (9.6)	16 (5.7)
	- (%)	145 (47.4)	115 (37.6)	108 (39.1)	129 (46.7)	124 (44.1)	114 (40.6)
	
	**p value**	**ns**		**ns**		**ns**	

EGFR	+ (%)	4 (1.3)	7 (2.3)	4 (1.5)	6 (2.2)	6 (2.1)	3 (1.1)
	- (%)	166 (54.8)	126 (41.6)	118 (43.1)	146 (53.3)	145 (51.8)	126 (45.0)
	
	**p value**	**ns**		**ns**		**ns**	

P-cad	+ (%)	42 (13.8)	40 (13.1)	30 (10.9)	42 (15.2)	40 (14.3)	37 (13.2)
	- (%)	129 (42.3)	94 (30.8)	93 (33.7)	111 (40.2)	110 (39.3)	93 (33.2)
	**p value**	**ns**		**ns**		**ns**	

ns: not significant.

A statistically significant association was observed between the VDR-positive cases and ER-positive cases (p = 0.0002). Additionally, VDR-positive cases have also been significantly correlated with HER2-negative cases (p = 0.0238), but this is probably due to the low number of positive cases for HER2 in our series of mammary carcinomas. CYP27B1 expression presented no significant associations with any of the markers analyzed. PgR was the only marker that displayed an inverse correlation with CYP24A1: specifically, cases positive for PgR were mostly negative for CYP24A1 (p = 0.0485).

The series of 189 tumours with both components (carcinomas *in situ *and the corresponding invasive tumour) allowed the evaluation of the expression of the VDR, CYP27B1 and CYP24A1 simultaneously in the two types of tumours (Additional file 2: Table S2). The results obtained show that the three proteins (VDR, CYP27B1 and CYP24A1) display a statistically significant correlation of expression between the two sections (carcinomas *in situ *and the matching invasive tumour). Thus, positive cases in the *in situ *component are also positive in the invasive component and the same is observed for the negative cases.

### Expression of the VDR, CYP27B1 and CYP24A1 according to the type of breast lesion

**Figure 2:**
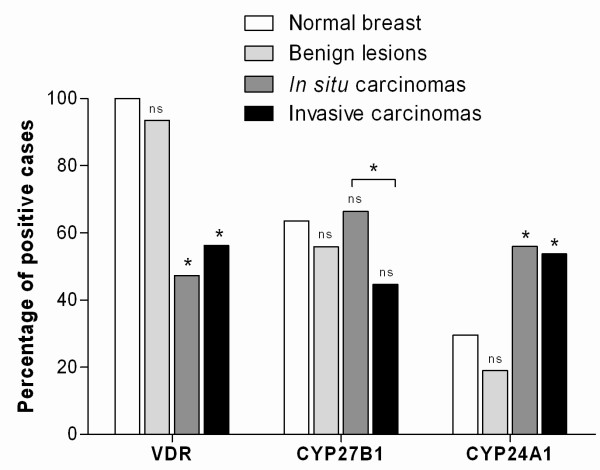
**Percentage of positive cases for VDR, CYP27B1 and CYP24A1 in the various types of breast samples studied**. Statistical analysis shown use normal breast as reference. An additional result is presented comparing the number of CYP27B1 positive cases between *in situ *and invasive carcinomas. (ns - not significant; * p < 0.05).

The frequencies of protein expression of the VDR, CYP27B1 and CYP24A1 in the different mammary tissues are shown in Figure 2. The normal mammary gland (29 cases), as expected, is positive for the expression of the VDR in all the cases studied (100%). The majority of the samples also displays immunostaining for CYP27B1 (63.6%) and, in contrast, the levels of expression of CYP24A1 are low (29.6%). The VDR is also highly expressed in benign lesions (93.5%) with a reduction in the percentage of positive cases in carcinomas *in situ *(47.3%) and in invasive carcinomas (56.2%). CYP27B1 expression does not vary greatly between the different breast lesions. However, between *in situ *and invasive carcinomas, a statistically significant decrease in the percentage of positive cases was observed (from 66.4% in carcinomas *in situ *to 44.6% in invasive carcinomas). In contrast, the expression of CYP24A1 is increased in carcinomas (56.0% in carcinomas *in situ *and 53.7% in invasive carcinomas) compared with the benign lesions (19.0%), which are mostly negative.

## Discussion

Vitamin D mediates anti-proliferative and pro-differentiation signalling in various epithelial tissues, including the mammary gland [6]. Therefore, it is reasonable to assume that disruption of the Vitamin D signalling and metabolic pathways may occur during tumour development. To explore this hypothesis, we have evaluated a cohort of 947 samples of human breast tissues for the presence of VDR, CYP27B1 and CYP24A1. Specifically, our series consisted of normal breast tissue (29 cases), preneoplastic benign mammary lesions (379 cases), carcinomas *in situ *(189 cases) and invasive breast carcinomas (350 cases). To the best of our knowledge, this is the first time that the expression of the VDR, CYP27B1 and CYP24A1 has been evaluated in histological sections of mammary lesions.

The three proteins have been found to be expressed in all breast tissues, although at different levels. VDR presented a nuclear localisation, as it would be expected for a nuclear receptor, while CYP27B1 and CYP24A1 enzymes displayed cytoplasmic staining with a granular pattern, which is consistent with their mitochondrial localisation. The immunohistochemical results were further validated and confirmed using quantitative real-time PCR and Western blotting.

Some studies have demonstrated that the VDR protein is expressed in samples from normal breast tissues and also in breast cancer biopsy specimens [14,15,24,25]. Our results have shown that the VDR is expressed in carcinomas. However, the percentage of positive cases that we have obtained (47.3% in carcinomas *in situ *and 56.2% in invasive carcinomas) is lower than the 80% to 90% that had been previously described in the literature [26,27]. This discrepancy can be explained by the development of new detection techniques and the use of different scoring methods. In this study, we have used the H-Score, the current method employed for other nuclear receptors, like ER [20], whereas in previous studies the presence of any staining was marked as positive. As far as we know, our study is the first to investigate the immunohistochemical expression of the VDR in a range of benign lesions and carcinomas *in situ *of the mammary gland. The percentage of positive cases for the VDR is higher in benign lesions than in invasive tumours (93.5% and 56.2%, respectively), while the carcinomas *in situ *display the lowest value of all (47.3%). There are some studies showing higher levels of VDR in tumour tissues [18,28], but this discrepancy can be attributed to the use of different evaluation techniques.

An interesting finding is the correlation between the expression of the VDR and the ER in both *in situ *and invasive carcinomas. In fact, the VDR is expressed in most ER-positive cases (54.7% in *in situ *carcinomas and 65.5% in invasive tumours). It is thought that one of the VDR functions is to counteract oestrogen-mediated proliferation and maintain differentiation [12]. Indeed, data support the concept that the anti-tumour effects of Vitamin D and its analogues on ER-positive human breast cancer cells are mediated through the down regulation of the ER itself and the attenuation of oestrogen responses, such as breast cancer cell growth [29,30]. Thus, being the VDR mostly expressed in ER-positive carcinomas, Vitamin D or its analogues may become an alternative therapy for these tumours in cases of resistance to ER-targeted therapy.

The levels of protein expression of CYP27B1 and CYP24A1 have not been previously studied in breast cancer. In colon cancer, a study using immunohistochemistry has demonstrated that CYP27B1 is present at equally high levels in normal colonic epithelium and colorectal cancer [31]. For CYP24A1 it has been shown that increasing amounts of this enzyme are present in normal colon tissue and pre-malignant lesions. In cancer, the expression of CYP24A1 decreases as a function of tumour cell dedifferentiation [32]. In breast tissues, McCarthy *et al*.[18] have demonstrated that CYP27B1 mRNA expression was significantly down regulated in adjacent non-cancerous tissue from women with breast cancer in comparison with individuals without cancer. Additionally, it has been shown that the expression of mRNA for CYP27B1 and the VDR was higher in carcinomas versus non-neoplastic tissue [17]. Considering differences in expression in benign and malignant breast tissues, we have observed an increased expression of CYP24A1 and a decreased expression of CYP27B1 with malignant progression. In fact, CYP27B1 was expressed in 55.8% of the preneoplastic lesions and this percentage is decreased in invasive tumours (44.6%), while carcinomas *in situ *display the highest value (66.4%) and these differences are statistically significant. In contrast, CYP24A1 is augmented more than 2.5 fold in invasive tumours (53.7%), compared with benign breast lesions (19.0%) and this difference is also significant (p < 0.0001). The *in situ *carcinomas exhibit the highest percentage of positive cases (56.0%). These observations are consistent with the results of Townsend and colleagues [17], which have demonstrated that there was an up regulation of CYP24A1 mRNA in breast tumour tissue, in comparison with normal breast. It has also been described that the *CYP24A1 *gene is amplified in breast cancer [33]. In contrast, another study has found no differences in the expression of the VDR, CYP27B1 and CYP24A1 mRNA in breast cancer and non-neoplastic mammary tissue [34]. These contradictory results may be explained by recent reports where it is described that VDR and CYP24A1 are under the post-transcriptional control of miRNAs [35,36].

Breast cancer is a process that evolves through the accumulation of (epi)genetic events that drive uncontrolled proliferation and resistance to apoptosis. The active form of Vitamin D is known for its capacity to modulate proliferation and induce apoptosis [6]. Consequently, malignant cells would need to develop mechanisms to deregulate Vitamin D metabolic and signalling pathways in order to allow tumour development [37]. Furthermore, it has been suggested that the Vitamin D produced in non-renal tissues is not released into the blood stream, but instead acts locally [38]. Therefore, the amount of Vitamin D available in the tissue depends on the relative amounts of CYP27B1 (synthesis) and CYP24A1 (catabolism). Accordingly, our results show a deregulation of these two enzymes in the different stages of breast carcinogenesis. The crucial step of transformation introduces a clear unbalance in the Vitamin D signalling and metabolic pathways. A reduction in the expression of the VDR in carcinomas indicates lower sensitivity of the tissue to Vitamin D control. Furthermore, a strong increase in CYP24A1 positive cases points to an enhanced ability of the cells to degrade this hormone. In contrast, the stable levels of CYP27B1 throughout the transformation process, with only a small decrease in invasive carcinomas, may reflect a lower capacity to metabolize Vitamin D into its active form.

## Conclusions

In summary, this is the first study to report the expression of the VDR, CYP27B1 and CYP24A1 in a series of normal breast, preneoplastic mammary lesions, breast carcinomas *in situ *and invasive tumours. We have correlated the expression of these Vitamin D partners with the expression of a panel of tumour biomarkers. Furthermore, we have confirmed these results by real-time RT-PCR. Overall, our results on the expression of the VDR, CYP27B1 and CYP24A1 suggest that there is a deregulation of the Vitamin D metabolic and signalling pathways in breast cancer, in order to favour tumour progression. Thus, during breast malignant transformation, tumour cells lose their ability to synthesize the active form of Vitamin D and to respond to Vitamin D effects, while increasing their ability to degrade this hormone.

## List of abbreviations

VDR: Vitamin D Receptor; TMA: Tissue Microarray; ER: oestrogen receptor; CK: Cytokeratin; EGFR: Epidermal Growth Factor Receptor; UDH: Usual Ductal Hyperplasia; ADH: Atypical Ductal Hyperplasia; CCL: Columnar Cell Lesions

## Competing interests

The authors declare that they have no competing interests.

## Authors' contributions

NL performed the practical work, analysed the data and drafted the manuscript. BS, DM and MG participated in the practical work. DV, LAV and FM analysed the data. JP and JLC designed the study and contributed to the manuscript. FS conceived the study, participated in its design and coordination, analysed the data and contributed to the manuscript. All authors read and approved the final manuscript.

## Pre-publication history

The pre-publication history for this paper can be accessed here:

http://www.biomedcentral.com/1471-2407/10/483/prepub

## Supplementary Material

Additional file 1 **Figure S1**: In MDA-MB-231 breast cancer cells CYP27B1 expression is induced by the treatment with PTH 100 nM for 4 h and CYP24A1 expression is induced by the treatment with Vitamin D (1,25(OH)_2_D_3_) 100 nM for 72 h. α-tubulin was used as a loading control Click here for file

Additional file 2 **Table S2**: VDR, CYP27B1 and CYP24A1 expression in tumours that display both the *in situ *and the invasive component in the same histological section. Click here for file
