# Supplementary figures and images for: Alterations in Vitamin D signalling and metabolic pathways in breast cancer progression: a study of VDR, CYP27B1 and CYP24A1 expression in benign and malignant breast lesions Vitamin D pathways unbalanced in breast lesions
Source: BMC Cancer. 2010 Sep 11;10:483. doi: 10.1186/1471-2407-10-483 (PMC2945944; doi:10.1186/1471-2407-10-483)

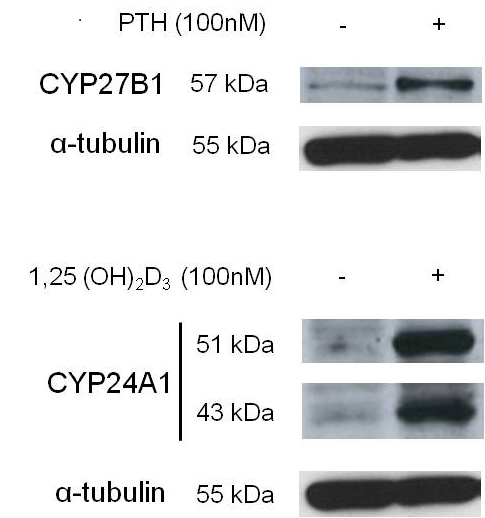

Supplement: Additional file 1 — Figure S1: In MDA-MB-231 breast cancer cells CYP27B1 expression is induced by the treatment with PTH 100 nM for 4 h and CYP24A1 expression is induced by the treatment with Vitamin D (1,25(OH)2D3) 100 nM for 72 h. α-tubulin was used as a loading control [file 1471-2407-10-483-S1.JPEG]
