# Supplementary material for: Alterations in Vitamin D signalling and metabolic pathways in breast cancer progression: a study of VDR, CYP27B1 and CYP24A1 expression in benign and malignant breast lesions Vitamin D pathways unbalanced in breast lesions
Source: BMC Cancer. 2010 Sep 11;10:483. doi: 10.1186/1471-2407-10-483 (PMC2945944; doi:10.1186/1471-2407-10-483)
Supplement: Additional file 2 — Table S2: VDR, CYP27B1 and CYP24A1 expression in tumours that display both the in situ and the invasive component in the same histological section. [file 1471-2407-10-483-S2.DOC]

**Additional file 2: Table S1 -** List of cases randomly selected for real-time PCR validation of the immunohistochemical results

|  | **Immunohistochemistry ^a^** | | | **Real-time PCR ^b^** | | |
| --- | --- | --- | --- | --- | --- | --- |
|  | **VDR** | **CYP27B1** | **CYP24A1** | **VDR** | **CYP27B1** | **CYP24A1** |
| Sample 1 | - ^c^ | + | - | 0.005  (0.004-0.006) | 0.005  (0.004-0.006) | undetectable |
| Sample 2 | - | + | + | undetectable | 0.285  (0.215-0.378) | nt |
| Sample 3 | - ^c^ | + | - | 0.018  (0.013-0.023) | 0.148  (0.105-0.209) | undetectable |
| Sample 4 | + | + | + | 0.55  (0.262-1.141) | 0.081  (0.032-0.205) | nt |
| Sample 5 | + | + | - | 0.169  (0.133-0.215) | 0.035  (0.027-0.045) | undetectable |
| Sample 6 | - ^c^ | - | - | 0.078  (0.020-0.307) | undetectable | nt |
| Sample 7 | - ^c^ | + | + | 0.0007  (0.0003-0.0019) | 0.006  (0.004-0.008) | 0.0001  (0.00009-0.00012) |
| Sample 8 | - ^c^ | + | + | 0.017  (0.015-0.020) | 0.116  (0.101-0.134) | 0.0003  (0.0001-0.0009) |
| Sample 9 | + | + | - | 0.225  (0.050-1.012) | 1.385  (0.936-2.049) | nt |
| Sample 10 | - ^c^ | + | + | 0.033  (0.013-0.080) | 0.052  (0.022-0.124) | 0.0006  (0.0003-0.0016) |

^a^ - Evaluated as described in the Methods section

^b^ - Normalized gene amount relative to GAPDH with respective range

^c^ - Although considered negative, positivity was observed, however the H-score did not cross the threshold established

nt - Not tested due to limited amount of biological material

**Additional file 2: Table S2 -** VDR, CYP27B1 and CYP24A1 expression in tumours that display both the *in situ* and the invasive component in the same histological section.

|  | | | ***In situ*** | | | | | | | | | |
| --- | --- | --- | --- | --- | --- | --- | --- | --- | --- | --- | --- | --- |
|  |  | | **VDR** | |  | | **CYP27B1** | |  | | **CYP24A1** | |
|  |  |  | + (%) | - (%) |  |  | + (%) | - (%) |  |  | + (%) | - (%) |
| **Invasive** | **VDR** | + (%) | 39  (35.1) | 5  (4.5) | **CYP27B1** | + (%) | 44 (39.6) | 10 (9.0) | **CYP24A1** | + (%) | 42 (44.2) | 8 (8.4) |
|  |  | - (%) | 10 (9.0) | 57  (51.4) |  | - (%) | 27 (24.3) | 30 (27.0) |  | - (%) | 11 (11.6) | 34 (35.8) |
|  |  | | p < 0.001 | |  | | p = 0.002 | |  | | p < 0.001 | |
